# Mechanosensitive Ion Channels and Neuroplasticity in Tuina Therapy: Molecular Mechanisms and Therapeutic Implications

**DOI:** 10.1155/np/1624832

**Published:** 2026-06-15

**Authors:** Mohammad Nasb, Dan Yang, Dandan Xu, Jing Zhou, Yan Zhao

**Affiliations:** ^1^ Rehabilitation Medicine Center/Tuina Department, Hubei Provincial Hospital of Traditional Chinese Medicine, Wuhan, 430061, China, hbtcm.edu.cn; ^2^ Hubei Shizhen Laboratory, Wuhan, 430061, Hubei, China; ^3^ Affiliated Hospital of Hubei University of Chinese Medicine, Wuhan, 430061, China, hbhtcm.com; ^4^ The First Clinical College, Hubei University of Chinese Medicine, Wuhan, 430065, China, hbtcm.edu.cn; ^5^ Hubei Province Academy of Traditional Chinese Medicine, Wuhan, 430061, China, hbtcm.edu.cn; ^6^ Hubei Key Laboratory of Theory and Application Research of Liver and Kidney in Traditional Chinese Medicine, Affiliated Hospital of Hubei University of Chinese Medicine, Wuhan, 430061, China, hbhtcm.com; ^7^ Department of Physical Therapy and Rehabilitation, Faculty of Health Sciences, Homs University, Homs, 010003, Syria

**Keywords:** complementary therapy, mechanosensitive ion channels, neuroplasticity, pain, traditional Chinese medicine, Tuina

## Abstract

Tuina is a traditional Chinese manual therapy distinguished from generic massage by its acupoint‐ and meridian‐based manipulations, and it is commonly used in the management of neurological and musculoskeletal disorders, although its underlying biological mechanisms remain incompletely defined. This review summarizes current experimental and clinical evidence on the neurobiological processes associated with Tuina therapy, with a focus on pain modulation and neural repair. Available evidence suggests that mechanical stimulation during Tuina may engage mechanosensitive ion channels, including transient receptor‐potential vanilloid (TRPV)1, transient receptor‐potential ankyrin 1 (TRPA1), and Piezo channels, thereby modulating nociceptive signaling, nitric oxide (NO)‐cyclic guanosine monophosphate (cGMP)‐protein kinase G (PKG) and toll‐like receptor 4 (TLR4)/nuclear factor kappa B (NF‐κB)‐related inflammatory pathways, Piezo1/Piezo2‐ and YAP/TAZ‐associated mechanotransduction, neurotransmitters and neuropeptides, and descending pain inhibitory circuits involving the periaqueductal gray (PAG)–rostral ventromedial medulla (RVM) system. In addition, emerging evidence indicates that Tuina may affect peripheral and central neuroplastic processes, including modulation of neuronal excitability, glial activity, and functional brain networks. While these findings provide a mechanistic framework for understanding reported clinical effects in conditions such as neuropathic pain (NP), low back pain (LBP), cervical disorders, and headache, heterogeneity in study design and intervention protocols limits definitive conclusions. Further well‐designed mechanistic studies and standardized clinical trials are required to clarify the role of Tuina in pain regulation and neuroplasticity and to support its evidence‐based application in clinical practice.

## 1. Background

The escalating global prevalence of chronic diseases, together with growing recognition of the need for complementary and integrative approaches alongside conventional pharmacological treatments, has stimulated renewed academic interest in complementary and alternative medicine therapies [1]. Among these, Tuina stands out as a particularly promising modality due to its unique blend of traditional wisdom and demonstrable therapeutic efficacy [2–4]. Tuina, a traditional Chinese medical massage, is an ancient therapeutic practice that has been used for thousands of years in China. As a core component of traditional Chinese medicine (TCM), Tuina involves a variety of manual techniques applied to specific acupoints, meridians, and affected areas of the body to restore balance, stimulate energy flow (Qi), and promote healing [5, 6]. Tuina should not be regarded as a generic form of massage. Rather, it is a structured TCM–based manual therapy in which treatment is guided by syndrome differentiation and delivered through predefined manipulative methods applied to selected acupoints, meridians and affected anatomical regions. This procedural specificity is reflected in the recently proposed STRICTOTM reporting guideline, which recommends explicit reporting of treatment rationale, manipulation techniques, treatment regimen, practitioner background, and comparator design, thereby recognizing Tuina as a reproducible therapeutic intervention rather than a nonspecific touch‐based exposure [7]. In contrast to massage approaches primarily organized around broad soft‐tissue relaxation, Tuina typically combines focal pressure, rhythmic rolling, kneading, pushing, grasping, plucking, joint mobilization, and traction according to both regional tissue findings and a diagnosis‐informed treatment strategy [7, 8]. This combination of anatomical targeting, acupoint selection and protocolized manual input is important because the biological effects of manual therapy are likely to depend not only on whether touch is delivered, but also on where, how and with what temporal pattern mechanical stimulation is applied. Focal and repetitive stimulation delivered to acupoint‐rich or symptomatic regions is likely to produce a more spatially restricted and biologically relevant afferent input than generalized massage strokes, with potential consequences for peripheral nociceptive processing, inflammatory signaling, and central pain modulation. Consistent with this view, recent preclinical studies indicate that Tuina can modulate mechanotransductive and nociceptive pathways in dorsal root ganglia, including Transient receptor‐potential vanilloid (TRPV)4–CaMKII signaling, while earlier studies also implicate TRPV1/transient receptor‐potential ankyrin 1 (TRPA1)‐related signaling and neuroinflammatory pathways. At the central level, resting‐state functional MRI data in painful cervical spondylosis suggest that Tuina is associated with altered activity in brain regions involved in pain processing and sensorimotor integration, supporting the possibility that structured peripheral mechanical input may contribute to neuroplastic changes relevant to symptom improvement [8, 9].

This distinction is also clinically relevant. Recent randomized trials have compared Tuina directly with physiotherapy or manual physical therapy rather than subsuming it within a generic massage category, indicating that Tuina is increasingly being evaluated as a discrete therapeutic modality with its own protocol content and treatment logic [10, 11]. A more defensible interpretation is that Tuina’s structured, diagnosis‐informed, and acupoint‐directed methods may generate a distinct pattern of mechanical stimulation capable of engaging mechanotransductive, inflammatory and neuroplastic pathways, thereby providing a plausible explanation for at least part of the clinical improvements discussed in this article. However, because many studies still incompletely report manipulation intensity, tissue depth, anatomical focus, and treatment dose, further standardized comparative and mechanistic studies remain necessary [7, 10].

Recent scientific research has begun to elucidate the underlying mechanisms by which Tuina exerts its therapeutic effects, providing increasing mechanistic and clinical evidence for its efficacy across a range of conditions [11]. A growing body of evidence has demonstrated Tuina’s analgesic properties, particularly in neuropathic pain (NP). Furthermore, Tuina’s analgesic effects may involve mechanosensitive, such as Piezo1 and Piezo2, which play a crucial role in pain perception [12, 13]. The relevance of mechanosensitive analgesia in Tuina depends not only on the molecular pathways discussed later, but also on the anatomical site and tissue depth at which stimulation is delivered. In contemporary Tuina studies, treatment is typically applied to the clinically affected surface and adjacent segmental tissues, for example, the cervical–shoulder region in neck pain and the lumbar–paraspinal region in low back disorders, rather than as a diffuse whole‐body massage [7, 8]. At these sites, manual stimulation is delivered to the skin, subcutaneous tissue, fascia, skeletal muscle, tendinous insertions, and periarticular soft tissues, where it is likely to recruit a mixed afferent population that includes low‐threshold cutaneous mechanoreceptors, proprioceptive endings in muscle–tendon units, and mechanically sensitive nociceptors [14, 15]. This anatomical specificity is important because the biological response to manual therapy depends on where mechanical force is applied, how deeply it is transmitted, and whether the stimulus is focal, rhythmic, compressive, or shear‐dominant [14]. Within this peripheral tissue environment, the receptor systems most relevant to the present review include Piezo channels and TRP‐family nociceptive channels. Piezo2 is strongly implicated in innocuous touch and proprioceptive mechanosensation, whereas Piezo2‐expressing nociceptors can also contribute to mechanical sensitization under pathological conditions; Piezo1 has been linked more broadly to mechanically responsive cellular signaling and tissue adaptation [15, 16]. In parallel, Tuina‐related preclinical studies suggest that manual stimulation can modulate TRPV1/TRPA1–nitric oxide (NO)–cyclic guanosine monophosphate (cGMP) signaling and TRPV4–CaMKII/CREB/NLRP3 signaling in dorsal root ganglia, consistent with reduced peripheral sensitization and nociceptor hyperexcitability [9, 17]. These findings make it more plausible that Tuina does not act through nonspecific touch alone but through targeted stimulation of anatomically and clinically relevant tissues whose afferent output converges on dorsal root ganglia, spinal nociceptive circuits and higher‐order pain networks [9, 14]. Accordingly, the analgesic effects described in this article are most reasonably interpreted as arising from a combination of site‐specific peripheral mechanotransduction and subsequent central modulation, rather than from generalized massage exposure [7–9, 16].

These findings suggest that Tuina can directly impact pain transmission and modulation atthea molecular level. Beyond pain relief, Tuina has shown significant rehabilitative effects, particularly in peripheral nerve injuries. Tuina has demonstrated the ability to promote the recovery of fine motor function and protect myelin integrity by influencing gene expression at the site of nerve injury [18, 19]. The therapeutic advantages of Tuina are multifactorial and align with key priorities in contemporary integrative medicine. As a nonpharmacological intervention, applying Tuina could minimize the risk of adverse drug reactions and reduce concerns related to long‐term medication dependency, rendering it a potentially safer complementary option for chronic disease management [2, 20, 21]. Its inherently patient‐centered nature allows for individualized treatment strategies that target both symptomatic relief and underlying pathophysiological imbalances [5]. Furthermore, Tuina offers a favorable cost–benefit profile compared to extended pharmacotherapy or surgical interventions, thereby enhancing accessibility, particularly in resource‐limited settings [22].

Additionally, Tuina employs a holistic, systems‐based approach that highlights the functional interdependence of body systems, potentially promoting systemic homeostasis and strengthening endogenous healing processes [23]. Within modern medical paradigms, the increasing body of evidence, such as randomized controlled trials (RCTs), systematic reviews, and meta‐analyses, continues to support Tuina’s clinical relevance and scientific credibility as an efficient and evidence‐based therapeutic modality. With a focus on its mechanisms and clinical applications, this review article attempts to give a thorough and current overview of the state of knowledge regarding Tuina therapy. This article aims to clarify the complex physiological and neurological pathways through which Tuina exerts its therapeutic effects and to consolidate the evidence supporting its efficacy by critically analyzing recent scientific literature, including RCTs, systematic reviews, and meta‐analyses.

## 2. Analgesic Mechanisms of Tuina Therapy

Despite having its roots in TCM, Tuina therapy is increasingly the focus of thorough scientific research intended to clarify its underlying analgesic mechanisms. Tuina exerts multifaceted effects on pain modulation, including the regulation of different biochemical mediators and intricate interactions between peripheral and central nervous system modulations.

### 2.1. Role of TRPV1/TRPA1 Channels, Piezo Channels, and Mechanotransduction

Mechanosensitive ion channels convert external mechanical stimuli into intracellular signals and represent an early event in the nociceptive pathway [24]. Tuina manipulations (pressing, kneading, and plucking) produce dynamic stresses on the skin and underlying tissues, thus triggering or blocking ion channel pathways and adjusting sensory inflow [9]. TRPV1 and TRPA1 are nonselective cation channels expressed in nociceptive afferents and are sensitive to noxious thermal, chemical, and mechanical stimuli [17]. Activation of TRPV1 and TRPA1 leads to Ca^2+^ influx, which can trigger NO synthase (NOS) and downstream NO–cGMP–protein kinase G (PKG) signaling, a pathway implicated in both pronociceptive and antinociceptive processes [25]. de Vente et al. [26] found that a single Tuina session substantially reduced the expression of NOS, soluble guanylate cyclase (sGC) β, cGMP, and PKG1 in the dorsal root ganglia while rising mechanical withdrawal threshold and thermal withdrawal latency in a rat model of mild chronic constriction injury. Inhibition of TRPV1 or TRPA1 with antagonists produced similar analgesic effects, suggesting that Tuina’s immediate analgesia is mediated by down‐regulating these nociceptor channels and the NO–cGMP–PKG cascade [17].

More specifically, Tuina therapy activates TRPV1 and TRPA1 channels, which sets off a series of events. These channels are essential nociceptors—specialized sensory receptors that identify and transduce noxious (painful) stimuli from the environment and internal tissues—rather than just passive ion conduits [27]. Pain signals are produced when they are activated by a variety of stimuli, such as mechanical force, which is relevant to Tuina’s manual techniques, and inflammatory mediators [28]. Therefore, Tuina’s ability to modulate these channels directly impacts the initial perception and transmission of pain signals, serving as a primary mechanism for its analgesic effects.

The activation of TRPV1 and TRPA1 channels by Tuina therapy induces a rapid influx of Ca^2+^ into neuronal cells. This Ca^2+^ influx activates downstream signaling pathways involved in nociception and inflammation, crucially involved in various cellular responses, including neuronal excitation, neurotransmitter release, and enzyme activation related to pain modulation and inflammation [5, 17, 29]. Previous studies underscore the significant role of TRPV1/TRPA1‐mediated Ca^2+^ influx in initiating nociceptive signaling cascades, thus substantiating their critical involvement in pain sensitization processes [3, 21]. When TRPV1/TRPA1 is activated, the amount of Ca^2+^ inside the cell increases, which activates NOS. NOS enzymes help change L‐arginine into NO, an element that acts as a messenger in many different ways and is crucial for numerous physiological processes, such as vasodilation, neurotransmission, and immune regulation [30]. In nociceptive pathways, NO has demonstrated dual functions: it can either facilitate or suppress nociception, depending upon its local concentration and the specific NOS isoform involved (nNOS, eNOS, or iNOS) [31, 32]. NO subsequently activates sGC, which helps change guanosine triphosphate (GTP) into cGMP. The rise in cGMP levels that follows this activates PKG, which is also called cGMP‐dependent protein kinase. This is an important part of cellular signaling [33–35]. It has been shown that activating PKG reduces the excitability of neurons, which leads to immediate pain relief that has been seen in clinical settings after Tuina therapy [17].

**Figure 1:**
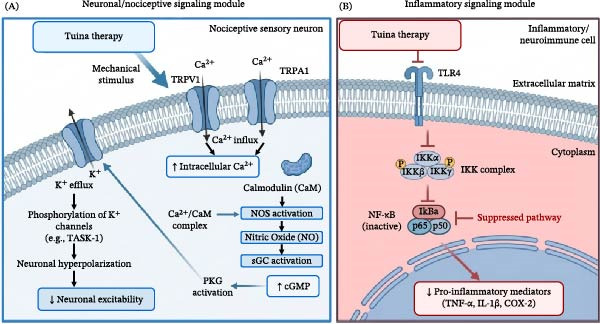
Conceptual schematic of Tuina‐related analgesic and anti‐inflammatory pathways across distinct pain‐related cell types. (A) In a nociceptive sensory neuron, Tuina‐associated mechanical stimulation may modulate TRPV1/TRPA1‐dependent Ca^2+^ influx, activate the NO–sGC–cGMP–PKG pathway, enhance K^+^ channel‐related hyperpolarization, and reduce neuronal excitability. (B) In a pain‐related inflammatory or neuroimmune cell, Tuina may suppress TLR4–IKK–NF‐κB signaling, thereby reducing pro‐inflammatory mediators such as TNF‐α, IL‐1β, and COX‐2. The schematic is intended as an integrative representation and does not imply that all depicted events occur within a single cell.

Furthermore, the activation of PKG inhibits IκB kinase (IKK) activity, thus suppressing the nuclear factor kappa B (NF‐κB) signaling pathway. NF‐κB is a key regulator of inflammatory responses, and its inhibition leads to decreased expression of pro‐inflammatory mediators, notably tumor necrosis factor‐alpha (TNF‐α), interleukin‐1 beta (IL‐1β), and cyclooxygenase‐2 (COX‐2), contributing significantly to the anti‐inflammatory and analgesic outcomes of Tuina therapy [36, 37]. Overall, the evidence supports that Tuina therapy effectively exerts analgesic and anti‐inflammatory effects primarily via the TRPV1/TRPA1‐mediated NO–cGMP–PKG signaling cascade. This mechanistic insight not only provides a scientific rationale for Tuina’s clinical efficacy but also highlights potential therapeutic targets for managing chronic pain and inflammation (Figure 1).

Mechanotransduction and piezo channels: Piezo1 and Piezo2 are major ion channels that are sensitive to mechanical stress and are found in numerous peripheral tissues. Piezo2 is responsible for noninvasive, gentle touch, proprioception, and the gut stretch reflex, while Piezo1 influences the formation of myelin and the tone of blood vessels. Research studies involving chronic compression of dorsal root ganglia (CCD) rats demonstrated that clockwise pressing and rubbing promoted Piezo2 expression while diminishing Piezo1 expression [13]. This differential regulation suggests that Tuina alters mechanotransduction from a nociceptive (Piezo1‐dominated) to a non‐nociceptive (Piezo2‐dominated) profile. Additional evidence is provided by a 2025 study that involved rats with sciatic nerve injuries, which demonstrated that Tuina decreased Piezo1 and Ca^2+^ levels while upregulating downstream transcriptional co‐activators YAP and TAZ, as well as myelin basic protein and neurofilament [12]. GsMTx4, a Piezo1 inhibitor, partially reversed these changes, confirming that Tuina promotes myelin repair and functional recovery through the Piezo1/YAP/TAZ pathway [12].

In visceral organs, long‐term abdominal‐and‐back Tuina upregulated Piezo2 and 5‐HT4 receptor mRNA and protein in diabetic rats, with concomitant increases in serotonin and 5‐HT4 receptor levels and improved gastric emptying [32]. These findings suggest that Tuina’s mechanical input is sensed by Piezo channels, which then modulate serotonergic signaling and smooth muscle contractility. Collectively, Tuina’s ability to attenuate nociceptive TRPV1/TRPA1 activity and to rebalance mechanosensitive Piezo signaling provides a mechanistic explanation for its rapid analgesic effects and its benefits in neuromuscular and gastrointestinal disorders.

### 2.2. Regulation of Inflammatory Mediators and Toll‐Like Receptor 4 (TLR4)‐Dependent Signaling

Pain signaling is closely coupled to inflammatory processes. Pro‐inflammatory cytokines, such as TNF‐α, IL‐1β, and IL‐6, sensitize nociceptors and upregulate ion channels like TRPV1 and TRPA1. Tuina’s analgesic efficacy, therefore, also depends on its capacity to modulate immune responses. TLR4/NF‐κB pathway. TLR4 recognizes pathogen‐associated molecular patterns and, through adapters, such as MyD88, IRAK1 and TRAF6, activates the NF‐κB and MAPK cascades, leading to the release of pro‐inflammatory cytokines. In spinal‐nerve‐ligation (SNL) and lumbar disc herniation (LDH) models, 14‐day Tuina interventions (pointing, stroking, and kneading) significantly reduced mRNA and protein levels of TLR4, IRAK1, TRAF6, TNF‐α, and IL‐6 [13, 25]. Intriguingly, injection of a TLR4 inhibitor mimicked Tuina’s effect, whereas a TLR4 activator blunted it [25]. Transcriptomic analyses of minor CCI rats showed that even a single Tuina session down‐regulated TLR and NF‐κB pathways [25].

MicroRNA‐mediated modulation: MicroRNAs fine‐tune gene expression in immune and neuronal cells. High‐throughput sequencing of dorsal‐root‐ganglion tissue after Tuina revealed 19 differentially expressed miRNAs associated with inflammation, with miR‐547‐3p identified as a key mediator [36]. Overexpression of miR‐547‐3p suppressed Map4k4 and downstream NF‐κB components (IκBα, p‐IκBα, and p65), reducing inflammatory cytokine production [36]. Similarly, upregulating miR‐146a in LDH rats attenuated TLR4 signaling and alleviated pain [37]. These findings suggest that Tuina exerts epigenetic control over inflammatory pathways. Suppression of pro‐inflammatory mediators. Clinical and pre‐clinical evidence shows that Tuina lowers systemic levels of TNF‐α, IL‐1, and IL‐1β in patients with LDH and reduces 5‐hydroxytryptamine (5‐HT) and prostaglandin E in sciatica [18]. In chronic constriction injury models, Tuina decreased IL‐6 in the serum and spinal cord while increasing the anti‐inflammatory factor SOCS3 [38]. It also downregulated Raf‐1–ERK–CREB signaling and phosphorylated MAPK, leading to a drop in IL‐1β expression and blockade of downstream inflammation [18]. Beyond NP, Tuina suppressed IL‐1β, IL‐17, and matrix metalloproteinases (MMP‐3 and MMP‐13) in knee‐osteoarthritis rats and lowered IL‐1β, IL‐6, TNF‐α, and PGE_2_ in lipopolysaccharide‐induced fever models [39, 40].

**Figure 2:**
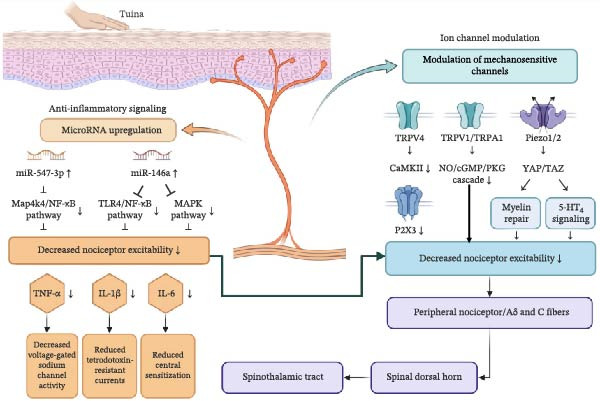
Peripheral initiation mechanisms of Tuina‐induced analgesia and their relationship to ascending nociceptive signaling. Mechanical stimulation during Tuina may modulate mechanosensitive ion channels, including TRPV1/TRPA1, P2X3, and Piezo1/2, together with downstream NO/cGMP/PKG and YAP/TAZ‐related signaling, thereby reducing nociceptor excitability. In parallel, Tuina may suppress TLR4/NF‐κB‐ and MAPK‐related inflammatory signaling, decrease pro‐inflammatory cytokines, and reduce peripheral sensitization. Together, these peripheral effects may reduce ascending nociceptive input transmitted by Aδ and C fibers to the spinal dorsal horn and spinothalamic tract.

These broader anti‐inflammatory effects may indirectly downregulate TRPV1 and TRPA1, since inflammatory mediators sensitize these channels. By integrating mechanical modulation of nociceptor ion channels with suppression of pro‐inflammatory signaling, Tuina therapy addresses both the physical and biochemical drivers of peripheral pain. The coordinated regulation of TRP/Piezo channels, TLR4‐dependent pathways and microRNA networks illustrates why Tuina can provide rapid relief while also promoting tissue repair. By addressing both the mechanical and chemical sources of pain, Tuina demonstrates a comprehensive approach to pain management. Mechanical stimulation during Tuina therapy engages mechanosensitive ion channels and suppresses inflammatory pathways (Figure 2), leading to decreased nociceptor excitability and reduced pro‐inflammatory cytokine release.

### 2.3. Neuroplasticity and Descending Pain Inhibition

Beyond its peripheral actions, Tuina also influences central nervous system processes, including neuroplasticity and the descending pain‐inhibitory system, to produce its analgesic effects. These central modulations are crucial for long‐lasting pain relief and functional recovery [41].

#### 2.3.1. Effects on Brain Functional Networks and Regional Homogeneity (ReHo)

Chronic pain frequently correlates with maladaptive neuroplastic alterations in the brain, influencing functional connectivity and regional brain activity. Functional magnetic resonance imaging (fMRI) studies indicate that Tuina therapy may modulate brain functional networks and ReHo, reflecting neuroplastic changes associated with pain alleviation and functional improvement [42]. Zhang et al. [43] examined the impact of Tuina massage therapy on the resting‐state brain functional network in patients with chronic neck pain. Their research demonstrated that Tuina therapy can influence the causal connections among intrinsic brain networks, indicating an alteration of connection between the anterior default mode network (aDMN) and the sensorimotor network (SMN) following 4 weeks of treatment [43]. This indicates that Tuina may reorganize brain activity patterns related to pain processing, resulting in a more adaptive functional state.

Song et al. [8] recently performed a resting‐state fMRI study on patients with painful cervical spondylosis, showing that Tuina therapy not only decreased visual analog scale (VAS) and neck disability index (NDI) scores but also led to changes in regional brain activity, particularly in ReHo values, across different brain regions. ReHo quantifies the synchronization of spontaneous neuronal activity within a specific brain region, with its variations frequently noted in chronic pain conditions. The changes in ReHo values seen after Tuina treatment show that Tuina may restore brain activity again to normal or change the way neurons operate collectively, thereby contributing to pain reduction and functional improvement [8]. These neuroimaging results offer substantial evidence of Tuina’s capacity to facilitate advantageous neuroplastic alterations in the brain, essential for enduring pain alleviation.

#### 2.3.2. Modulation of the Descending Pain Inhibitory System (DPIS) (Periaqueductal Gray [PAG]–Rostral Ventromedial Medulla [RVM])

The DPIS is a crucial neural pathway originating from the brainstem that modulates pain signals at the spinal cord level. Key structures involved in this system include the PAG and the RVM. Activation of the DPIS leads to the release of endogenous opioids and other neurotransmitters, resulting in pain inhibition [4].

**Figure 3:**
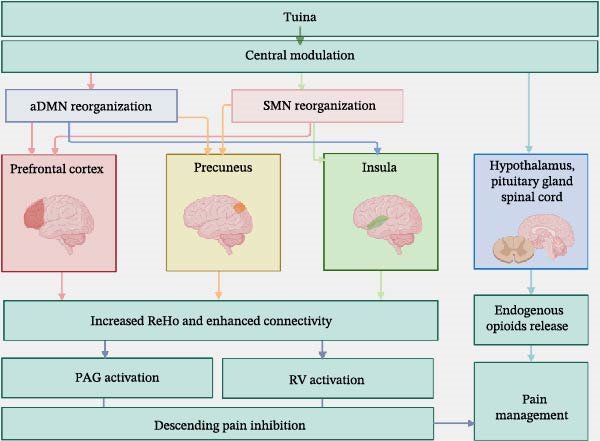
Central effects of Tuina therapy on brain networks and descending pain inhibition. Tuina induces reorganization of the anterior default mode network (aDMN) and sensorimotor network (SMN) connections. This reorganization is associated with increased regional homogeneity (ReHo) in areas such as the prefrontal cortex, precuneus, and insula, contributing to pain relief. Furthermore, Tuina effects involve modulation of endogenous opioids and the reduction of ReHo in pain‐related regions, including periaqueductal gray (PAG) and rostral ventromedial (RV).

While direct studies specifically on Tuina’s modulation of the PAG–RVM pathway are still emerging, the observed central effects of Tuina, particularly its influence on brain functional networks and its analgesic efficacy, strongly suggest its involvement. Other TCM modalities, such as acupuncture, have been shown to activate the DPIS, and given the shared theoretical underpinnings and some overlapping mechanisms with Tuina, it is plausible that Tuina also engages this system [44]. Further research is needed to directly investigate the modulatory implications of Tuina on the RVM and PAG, which have critical roles in the DPIS [4]. Figure 3 illustrates how Tuina therapy reorganizes intrinsic brain networks, normalizes ReHo and activates the DPIS via endogenous opioid release.

### 2.4. Neurotransmitter and Neuropeptide Regulation

Tuina’s analgesic effects are also mediated by its influence on various neurotransmitters and neuropeptides, which play crucial roles in pain transmission, modulation, and overall neurological function.

#### 2.4.1. Influence on Serotonin (5‐HT), Endorphins, and Other Neurotransmitters

Neurotransmitters, such as serotonin (5‐HT), endorphins, and gamma‐aminobutyric acid (GABA), are intimately involved in pain pathways and mood regulation. Tuina therapy has been shown to modulate the levels and activity of these neurochemicals, contributing to their analgesic and psychological benefits. Wang et al. [45] conducted a systematic review and meta‐analysis on the effect of Tuina on sleep quality, psychological state, and neurotransmitter levels in patients with insomnia.

Tuina has also been found to possess a regulatory effect on the hypothalamic–pituitary–adrenal (HPA) axis. A systematic review of Tuina as a treatment for insomnia reported the activation of corticotropin‐releasing hormone (CRH)/CRH receptor 1 signal pathway in the hypothalamus, which in its turn stimulated the HPA axis [46, 47]. The activation changed the main neuroendocrine mediators, such as adrenocorticotropic hormone (ACTH), cortisol, 5‐HT, and GABA. These findings indicate that Tuina modulates stress‐related neuroendocrine pathways and inhibits neurotransmitters. It is worth noting that 5‐HT levels were greatly elevated with Tuina, which led to enhanced sleep quality, decreased anxiety, and depressive symptoms, as well as enhanced pain control, due to the effects of serotonin in the descending pain inhibitions and care of sleep–wake cycles [45, 48]. Moreover, the general understanding of manual therapies and TCM suggests that Tuina may stimulate the release of these endogenous opioids, contributing to its pain‐relieving effects. Preclinical studies suggest that endogenous opioid peptides constitute an important basis of Tuina‐induced analgesia [49].

More research is needed on the different kinds of endorphins and how they influence the response to Tuina. Tuina has also been demonstrated to affect various neurochemicals. For instance, a study on abdominal Tuina in rats with hypoxic‐ischemic brain injuries showed that the hippocampus has higher levels of 5‐HT 1A receptor (5‐HT1A R) and synapsin‐1 (Syn1), which suggests that there are more neurochemical changes than just those which occur in pain pathways [50]. A separate investigation showed that Tuina therapy might help with pain relief by breaking down neurotransmitters like 2‐picolinic acid, 5‐hydroxy‐tryptophan, glutathione, and betaine‐aldehyde [51]. The findings collectively illustrate the complex neurochemical context affected by Tuina, which contributes to its multiple therapeutic effects in pain relief and neurological disorders. Table 1 shows a summary of the latest research that shows how effective Tuina is as a treatment for different neurological conditions along with how it operates.

**Table 1:** Summary of therapeutic effects and mechanisms of Tuina therapy in neurological diseases and symptoms.

Disease/symptom	Effect of Tuina	Key findings	Tuina intervention details	Reference
Peripheral nerve injury (PNI)	Alleviates pain, preserves motor function, and modulates pain‐related brain and motor cortex activity	In rats after sciatic nerve transection and repair, Tuina applied to the gastrocnemius muscle reduced pain, preserved motor function, and was associated with adaptive brain plasticity	Method: emulator‐based twirling and kneading, 0.45 N, 60 times per min, 10 min daily, from postoperative day 7 for 4 months. Location: right gastrocnemius muscle of the injured hindlimb	[52]
Peripherally‐induced neuropathic pain (pNP)	Analgesic; reduces inflammation; modulates ion channels; inhibits glial activation; and normalizes brain function	This review links Tuina analgesia in peripherally induced neuropathic pain to reduced inflammation, ion channel modulation, inhibition of spinal glial activation, and normalization of brain functional changes	Method: rubbing, kneading, and pressing; some studies used pointing, stroking, and kneading or clockwise pressing and rubbing	[13]
Insomnia, anxiety, depression	Improves sleep quality, relieves anxiety and depression, and regulates neurotransmitter levels, including serotonin	Tuina for insomnia was associated with better overall clinical response, higher serotonin levels, lower sleep, anxiety, and depression scores, and only minor rarely reported adverse events	Tuina involves manual techniques such as pressing, rubbing, pulling, and pinching, but no single standardized protocol was specified	[45]
Peripheral nerve injury (PNI)	Promotes recovery and improves symptoms of damaged nerves	Tuina may aid peripheral nerve injury recovery through autophagy, synaptic plasticity, axonal regeneration, and remyelination, with some studies also indicating adaptive somatosensory cortical changes after sciatic nerve injury	Not specified in source (review‐level synthesis; no single standardized protocol reported)	[18]
Neuropathic pain	Alleviates neuropathic pain by inhibiting microglial activation and reducing secretion of inflammatory cytokines in the spinal cord	In chronic constriction injury rats, Tuina showed time‐dependent cumulative analgesia with reduced markers of activated microglia and lower tumor necrosis factor alpha and interleukin 1 beta in the spinal dorsal horn	Thumb pressing and rubbing at ipsilateral Chengshan (BL57), 5 N at 2 Hz for 10 min, starting day 4 after surgery for 10 days, on the posterior lower leg/gastrocnemius region	[53]
Cervical spondylosis (neck pain and brain activity)	Alleviates pain and improves cervical spine dysfunction, with associated modulation of brain regional activity	After 2 weeks of treatment, pain intensity and neck disability scores decreased, and resting‐state functional magnetic resonance imaging showed altered regional homogeneity in multiple pain‐related and default‐mode network regions	Manual pressure, vertical flicking, kneading, and sustained acupressure were applied to neck and scapular acupoints for 15–20 min, 2–3 times weekly for 2 weeks (6 sessions). Sites: neck, shoulder, and scapular regions; points EX‐HN14, GB20, GB12, SI12, SI13, GB21, and SI11	[8]
Chronic nonspecific neck pain	Greater improvement in pain and function when combined with Yijinjing exercise than with Tuina alone	In an RCT of 102 patients, Tuina plus Yijinjing improved pain, neck disability, anxiety, and trapezius hardness more than Tuina alone at 8 weeks, with benefits maintained at 12‐week follow‐up	Tuina was given 3 times weekly for 8 weeks (24 sessions) using soft tissue, acupoint, and spinal manipulation techniques. Sites: neck and shoulder soft tissues and cervical spine; acupoints GB20, DU16, GB21, SJ14, and SI14	[54]
Lumbar disc herniation (LDH)	Evaluated as part of a planned multicentre randomized controlled trial protocol; clinical efficacy outcomes are not yet reported in this protocol article	This source is a trial protocol, not an outcomes study, for a multicentre RCT comparing traditional Chinese exercises alone vs Tuina plus exercise, with ODI as the primary outcome and pain, quality of life, and gait as secondary outcomes	Tuina included local muscle release and lumbar joint adjustment with rubbing, kneading, acupoint pressing, oblique‐pulling, and posterior extension for 20–30 min. Sites: lumbar spine and buttocks; points included BL23, BL24, BL25, BL40, and GB30	[55]
Diabetic peripheral neuropathy (clinical)	Improves clinical symptom scores and some nerve conduction measures.	A meta‐analysis of 24 RCTs (1989 participants) found that Chinese Tuina improved Toronto Clinical Scoring System scores and selected nerve conduction velocities	Meta‐analysis showed Tuina was mainly applied to the limbs using loosening techniques. Sites included limb points ST36, SP6, KI3, LI11, KI1, BL57, BL40, and GB34, with some studies also using abdominal points CV12, CV4, CV6 and back points BL13, BL20, and EX‐B3	[3]
Peripheral neuropathic pain (animal studies)	Promotes nerve repair by improving functional recovery and central plasticity after peripheral nerve injury	In rats after sciatic nerve transection and repair, Tuina applied to the gastrocnemius improved behavioral recovery and altered brain activation patterns, suggesting adaptive brain plasticity	Emulator‐simulated twirling and kneading were applied for 10 min daily from postoperative day 7 for 4 months to the gastrocnemius of the injured hindlimb	[52]
Sciatic nerve injury/muscle atrophy (animal)	Prevents or reduces muscle atrophy and improves microcirculation, linked to phosphoinositide 3‐kinase and protein kinase B pathway signaling changes	In a rat model of sciatic nerve injury, Tuina reduced muscle atrophy, improved muscle ultrastructure and microcirculation, and modulated IGF‐1/PI3K/Akt signaling in the anterior tibial and soleus muscles	Tuina used simulator‐delivered finger pressing, plucking, and kneading at 4 N and 60 cycles/min; each point was treated for 3 min (9 min total) over 20 sessions with a rest day after 10. Sites: operated‐side lower limb at BL37, BL57, and GB34 along the sciatic nerve pathway	[56]
Hypoxic‐ischemic brain injury (neonatal, animal)	Improves behavioral outcomes and is associated with hippocampal changes in serotonin receptor and synaptic protein measures	In neonatal rats with hypoxic–ischemic brain injury, abdominal Tuina was given for 28 days starting 24 h after modeling and was assessed for behavioral outcomes and hippocampal 5‐HT1A receptor and synapsin‐1 expression in the CA1 region	Abdominal Tuina was applied to the abdomen, targeting the conception vessel, kidney, spleen, and stomach meridians and related front‐Mu points	[50]

## 3. Clinical Applications of Tuina in Neurological and Musculoskeletal Disorders

Tuina therapy has demonstrated clinically relevant benefits across a wide spectrum of neurological and musculoskeletal disorders, offering a valuable therapeutic option for patients suffering from various painful and debilitating conditions. The versatility of Tuina techniques allows for tailored interventions that address the specific pathologies and symptoms associated with these disorders. This section will explore the evidence supporting Tuina’s application in key clinical areas.

### 3.1. Tuina for NP Conditions

NP, characterized by pain arising from damage or disease affecting the somatosensory nervous system, is often challenging to treat with conventional methods. Tuina has emerged as a promising nonpharmacological intervention for various NP conditions, with growing evidence supporting its analgesic and rehabilitative effects.

#### 3.1.1. Peripheral Neuropathy

Diabetic peripheral neuropathy (DPN) is a prevalent and debilitating complication of diabetes, causing symptoms that involve numbness, paresthesia, and pain. Yan et al. [3] concluded that Chinese Tuina therapy is a safe and effective approach to treat DPN. The research demonstrated that Tuina can substantially reduce clinical symptoms and enhance motor nerve conduction velocity (MNCV) and sensory nerve conduction velocity (SNCV) in particular nerves, including the common peroneal nerve, sural nerve, and ulnar nerve [3]. This implies that Tuina not only alleviates symptoms but also supports the functional recovery of impaired peripheral nerves in patients with diabetes. In the included DPN trials, Tuina was applied primarily to the limbs using loosening‐type manipulations, with some studies combining acupoint Tuina with meridian patting; commonly used acupoints included Zusanli (ST36), Sanyinjiao (SP6), Taixi (KI3), Quchi (LI11), Yongquan (KI1), Chengshan (BL57), and Weizhong (BL40), with additional abdominal (e.g., CV12, CV4, and CV6) and back (e.g., BL13, BL20, and EX‐B3) points reported in some trials [3].

#### 3.1.2. Radiculopathy

Radiculopathy, which occurs when spine’s nerve roots are compressed, usually manifests as pain, numbness, or weakness in the limbs. Tuina has been extensively utilized for cervical and lumbar radiculopathy, with research demonstrating its efficacy in enhancing the symptoms and functional outcomes. Ding et al. [57] demonstrated that novel Tuina manipulations were superior in ameliorating functional symptoms in patients with cervical spondylosis of the vertebral artery type, as compared to conventional Tuina manipulations.

Although the study did not reveal significant alterations in cerebral blood flow, the enhancement of functional symptoms underscores Tuina’s direct influence on patient well‐being. Moreover, Song et al. [8] illustrated that Tuina therapy not only diminished pain and disability scores (VAS and NDI) in individuals with painful cervical spondylosis but also elicited advantageous alterations in regional brain activity, as assessed by ReHo values. This suggests that Tuina’s effects go beyond merely relieving local symptoms and also affect the manner in which pain is processed in the brain. Researchers have shown that Tuina, especially when combined with complementary therapies such as acupuncture, may be superior to standard treatments for relieving symptoms and improving functional status in cases of lumbar radiculopathy, which is often caused by LDH [18]. These findings underscore Tuina’s significance in addressing the complex relationship of mechanical compression, inflammation, and neurological dysfunction in radiculopathy. In vertebral‐artery‐type cervical spondylosis studies, Tuina was delivered as “innovative” versus routine textbook‐based manipulations (protocol details not reported in the abstract) [57], whereas in the rs‐fMRI cohort of painful cervical spondylosis, Tuina comprised standardized neck/shoulder and scapular soft‐tissue techniques with acupoint pressure at Jingbailao (EX‐HN14), Fengchi (GB20), Wangu (GB12), and acupressure at Bingfeng (SI12), Quqiguan (SI13), Shoulder Well (GB21), and Tianzong (SI11) (15–20 min, 2–3 times weekly for 2 weeks) [8].

### 3.2. Tuina for Pain Syndromes

Pain syndromes are among the most common reasons for seeking medical attention, significantly impacting the quality of life. Tuina, with its direct application to muscles, tendons, ligaments, and joints, is particularly well‐suited for treating these conditions.

#### 3.2.1. Low Back Pain (LBP) and LDH

LBP is a pervasive global health issue. Tuina has been extensively studied for its efficacy in managing LBP, including that caused by LDH. A systematic review by Kong et al. [58] suggested that Tuina‐focused integrative Chinese medical therapies (TICMT) might be effective complementary and alternative treatments for inpatients with LBP, especially when combined with Chinese herbal medicine or acupuncture. The review indicated statistically significant effects on pain and functional status. These findings are further supported by a bibliometric analysis by Xu et al. [59], which identified LBP as a new research frontier in Tuina for chronic pain management, highlighting the growing interest and evidence in this area. In Tuina‐focused integrative LBP protocols summarized in the inpatient systematic review, Tuina is described as combined soft‐tissue manipulation (including stroking, kneading, and percussion) with spinal manipulation/mobilization (with or without thrust), typically delivered for about 20–30 min per session with a variable number of sessions across trials [58].

#### 3.2.2. Neck Pain and Cervical Spondylosis

A common musculoskeletal disorder is neck pain, which is often caused by cervical spondylosis. Cervical spondylosis is a chronic degenerative condition of the cervical spine, affecting 3.3 patients per 1000 in the general population [60]. Tuina has demonstrated efficacy in ameliorating functional symptoms associated with cervical spondylosis [8]. The neuroimaging studies also show that Tuina affects brain activity that is linked to chronic neck pain, which suggests that central mechanisms play a role in its therapeutic effects [43]. These studies collectively suggest that Tuina may assist people with neck pain and improve their quality of life. The chronic neck pain neuroimaging study assessed symptom scores before and after 4 weeks of Tuina therapy (specific techniques not reported in the abstract) [43], while the painful cervical spondylosis rs‐fMRI study standardized an acupoint‐guided protocol targeting neck/shoulder and scapular tissues with named cervical/shoulder acupoints and a defined dose schedule (15–20 min, 2–3 times weekly for 2 weeks) [8].

#### 3.2.3. Tension‐Type Headache (TTH) and Migraine

Headaches, particularly TTH and migraine, are common neurological disorders that significantly impact daily life. Headache is estimated to affect around 50% of the general population [61]. Fan et al. [62] conducted a systematic review and meta‐analysis on the effectiveness and safety of Tuina for TTH, concluding that Tuina has a certain effect in treating TTH, showing superiority over drugs in improving the effectiveness rate and reducing pain intensity. The study noted that adverse events were tolerable. While the evidence for migraine is still developing, the mechanisms of Tuina, particularly its effect on neurotransmitters and pain pathways, suggest its potential beneficial effect in managing migraine symptoms as well [62].

In the TTH meta‐analysis, Tuina is described as a therapeutic manipulation that may include soft‐tissue relaxation techniques (e.g., poking‐channel, kneading, and rolling) followed by bone‐setting manipulation, although specific treatment sites and acupoints were not reported in the abstract [62], and the migraine–TTH review provides no intervention‐specific Tuina protocol details [61].

## 4. Safety Profile and Reported Adverse Events

Because Tuina therapy is increasingly integrated into modern clinical practice, its safety profile, clinical effectiveness, and quality of supporting evidence warrant careful and systematic evaluation. While traditional practice has largely relied on empirical experience, contemporary clinical application requires rigorous assessment under evidence‐based medicine frameworks, particularly with respect to adverse event reporting and methodological transparency. Current evidence suggests that Tuina therapy is generally well tolerated, with most reported adverse events being mild and transient. For example, Liu et al.’s [2] systematic review of Tuina for chronic ankle instability reported no major safety concerns, supporting an overall favorable safety profile. Similarly, Fan et al. [62] noted that adverse effects associated with Tuina in TTH were tolerable. In the context of insomnia, a systematic review and meta‐analysis of 23 RCTs reported that only two trials documented adverse reactions; where reported, these events were mild, primarily including drowsiness, pruritus, and malaise, and no serious adverse events were observed [45].

Evidence from musculoskeletal conditions further supports this safety pattern, although it also highlights important limitations in reporting. In chronic nonspecific LBP, one RCT documented four adverse events across study arms, with only a single transient episode of low back pain occurring after Tuina therapy in the combined treatment group, which required no intervention [11]. A PRISMA‐compliant systematic review and meta‐analysis in the same clinical context similarly found that only six included randomized trials reported adverse events, none of which were serious [21]. However, a separate outcomes‐reporting systematic review emphasized that safety monitoring and adverse event reporting were frequently inadequate across Tuina trials, indicating potential underreporting and methodological inconsistency [63]. For joint‐related disorders, a randomized crossover trial in patients with knee osteoarthritis reported no serious adverse events during either the Tuina intervention or the comparator (health education) period [20]. Likewise, a recent meta‐analysis of Tuina for chronic ankle instability reported that adverse events were described in seven included trials; apart from one event in a control group that resolved spontaneously within 48 h, no other adverse events were reported, and no significant differences were observed between intervention and control groups [2].

From a clinical perspective, Tuina is a nonpharmacological and noninvasive manual therapy, which avoids risks associated with drug‐related adverse effects or invasive procedures [2, 63]. Nevertheless, its safety remains dependent on appropriate practitioner training, adherence to contraindications, and individualized treatment planning. Improper technique or inadequate patient assessment may increase the likelihood of adverse outcomes, although such events are infrequently documented in the current literature. Importantly, the consistently low rate of reported adverse events across studies should be interpreted with caution due to heterogeneity in reporting standards and the frequent absence of structured safety monitoring protocols [20, 21].

## 5. Conclusion and Future Perspective

The growing body of evidence supporting Tuina’s efficacy and safety has significant implications for clinical practice. Tuina has the potential to be adopted as a beneficial adjunctive or alternative treatment for patients with chronic pain and neurological impairments, especially when first using nonpharmacological treatments or experiencing adverse effects of standard methods. It is a patient‐centered intervention since it is holistic and can lead to an improvement in overall well‐being because it can be adjusted to meet the needs of a specific patient. Tuina ought to be considered to be an effective method of treatment by clinicians particularly when it has shown good evidence for example chronic musculoskeletal pain and some forms of neuropathies. Although the results are promising, there are a number of areas that could be explored. Quite a number of studies failed to specify the techniques of Tuina (e.g., pushing, kneading, and rolling), the intensity of manipulation, or the focus of the treatment on any of the anatomies. Also, the length and the number of sessions of treatment were very uneven, and they are not well documented. Such methodological discrepancies do not support replication and comparison between studies. The problem is the constant demand of high‐quality RCTs with bigger sample sizes, rigorous methods and long‐term follow‐ups to reinforce the evidence base.

The clarification of the exact mechanisms of action, especially the central nervous system changes and neurochemical alterations, should also be in the future studies through the application of the superior neuroimaging and neurochemical methods. Research with comparative effectiveness whereby Tuina is compared to or used together with other conventional and complementary therapies would be informative to clinical decision‐making. Moreover, the studies of standardization of Tuina techniques and the introduction of objective outcome measures would help popularize it and implement it in mainstream healthcare systems. Tuina therapy can have an even more important role in world health and pain management by filling the gap between traditional practice and modern biomedical science.

## Author Contributions

**Mohammad Nasb**: conceptualization, writing – original draft, visualization, writing – review and editing. **Dan Yang**: writing – original draft, visualization, writing – review and editing. **Dandan Xu**: writing – review and editing. **Jing Zhou**: conceptualization, supervision. **Yan Zhao**: conceptualization, supervision, funding acquisition.

## Funding

This work was supported by the State Administration of Traditional Chinese Medicine (Grant GZY‐KJS‐2025‐008).

## Disclosure

After using the ChatGPT, authors reviewed and edited the content as needed and take full responsibility for the content of the published article.

## Ethics Statement

This article does not contain any studies with human or animal subjects.

## Consent

This article does not contain any individual person’s data in any form.

## Conflicts of Interest

The authors declare no conflicts of interest.

## Data Availability

As this article is a narrative review, no data were used or analyzed in this study.
